# Molecular docking analysis of imiquimod with the TGF-β targets for oral carcinoma

**DOI:** 10.6026/97320630019467

**Published:** 2023-04-30

**Authors:** Jayanthi Pazhani, Vishnu Priya Veeraraghavan, Selvaraj Jayaraman

**Affiliations:** 1 Centre of Molecular Medicine and Diagnostics (COMManD), Department of Biochemistry, Saveetha Dental College and Hospitals, Saveetha Institute of Medical and Technical Sciences, Saveetha University, Chennai-600077, India

**Keywords:** Oral cancer, TGF-β signaling, Imiquimod, molecular docking

## Abstract

TGF-βsignalling pathway is the main signalling pathways that regulate various biological functions such as cell proliferation, apoptosis, metabolic dysregulation, and metastasis in many cancer cells. Previous studies have elaborated the role of TGF-βsignalling targets have a significant regulatory function in various cancers. Moreover targeting the epithelial to mesenchymal transition markers in oral squamous cell carcinoma not yet elucidated. Therefore, it is of interest to document the molecular docking analysis of TGF-βsignalling pathway targets such as Smad2, GATA2 and MAFG with imiquimod. These results suggest that targeting the TGF-βsignaling pathway downstream targets with imiquimod might leads to an improved outcome as potential therapeutic options in oral carcinoma.

## Background:

Oral carcinoma is the sixth most common cancer in the world on accounting for about 2 - 4 % of all other cancers and it creates a major public health burden [1]. The occurrence of oral squamous cell carcinoma was significantly augmented globally around 650, 000 new cases and 350, 000 cancer mortality on each year [2]. This oral cancer and their categories like Head and neck carcinoma, tongue carcinoma were accounts for 50 - 70 % of total cancer mortality and also have the highest death incidence in Asian countries [3]. In detail, oral carcinoma is the most common cancer in South East Asia (India, Bangladesh, Pakistan, and Sri Lanka) where it also records for one-third of various cancers [4]. The main factors for causing OSCC due to environmental, lifestyle, and genetic factors causing malignant advanced stage is known [5].

Imiquimod, 1 H-imidazole-[4,5-c] quinolines is a synthetic compound which that belongs to the nucleoside analogue of imidazoquinolinone family known for its competence as immune response modifiers and stimulators [6]. Majorly, biological effects of imiquimod drug mainly show efficient activities which are the induction of endogenous antiviral pro-inflammatory mediators [7]. In addition, to the antiviral activity, imiquimod has exposed a very exciting efficacy in contradiction of several types of tumors, thus increases the research about its peculiar mechanism of action and its potential antitumor applications [8]. In past, SchÖn MP and SchÖn M studies stated that imiquimod increases the expression of the death receptor CD 95 (Fas) and its binding to the Fas-ligand, resulted cell death in tumor cells and also it significantly reduces the expression of the anti-apoptotic Bcl-2 protein [9]. Moreover, extended study of his research group stated that the imiquimod significantly resulted the release of mitochondrial cytochrome c and activation of caspase-9 and terminal caspases, mainly caspase-3, thus causing apoptosis of the malignant cells [10].

In current years, TGF-β pathway has shed into light due to its pleiotropic role in regulating cell growth, differentiation, apoptosis, and motility at the tumor level, and also regulates various biological processes in extracellular matrix production, angiogenesis, and cellular immune response at the tumor microenvironment level [11]. The results indicated that imiquimod might be a therapeutic target for TGF-β signaling which regulates the invasion and metastasis in tumors. In Oral Squamous Cell carcinoma, targeting the TGF-β pathway were poorly understood. Molecular docking is a computational process that visuals spatially dock a smaller molecule into a macromolecular and can score the complementary value at the exact binding sites, which are mainly used for structure-based drug design [12]. In this study, we explored the molecular mechanistic action of imiquimod with TGF- β signaling targets (Smad2, GATA2, and MAFG) using molecular docking [13]. Overall, we found that imiquimod has a significant binding affinity with TGF- β targets which provide a therapeutic targeting option for oral cancer treatment.

##  Materials and Methods:

## Protein preparation:

The crystal structure of TGF-β signaling targets such as Smad2, GATA2 and MAFG (Smad2 - PDB ID: IKHX; GATA2 - PDB ID: 509B; MAFG - PDB ID: 7X5E) was retrieved from RPSC Protein Data Bank (http://www.rcsb.org/) for molecular docking studies. Chain A of the respective proteins was used for macromolecule preparation. The coordinates of PDB structures were prepared for molecular docking by removing the water ions and ligands using Python molecule viewer. The drug ability pocket prediction was performed for the proteins using PockDrug-server.

## Ligand preparation:

Imiquimod, 1 H-imidazole-[4,5-c] quinolines, a synthetic compound was selected for this study. The 3D structures of these compounds were obtained from Pubchem. The drug-like properties of the molecules were determined using SWISS-ADME prediction. The avogadro server was used to minimise energy of synthetic compounds and 3D coordinates were prepared. The geometry of built synthetic drug, imiquimod was enhanced, partial charges were also calculated, and saved as mol2 files that was passed, as usual, to ADT for pdbqt file preparation.

## Molecular docking procedure:

The active site residues in the substrate-binding domain (SBD) of Smad2, GATA2 and MAFG were retrieved from the literature. The active site amino acids of targets were determined using Discovery studio 4.5. AutoDock tool was utilized to generate grids, calculate dock score and evaluate the conformers of compounds bound in the SBD of Smad2, GATA2 and MAFG. The grid maps were centred with box size of 50 x 50 x 50 xyz points at an active site residues of the proteins Smad2, GATA2, and MAFG were generated with AutoGrid. As per genetic algorithm, all the torsions could rotate during docking analysis. The Lamarckian genetic algorithm and the pseudo-Solis and Wets methods in molecular docking analysis were applied mainly for minimization, and also for using default parameters.

##  Results and Discussion:

Auto Dock 4.2, was regarded as the finest docking technique mainly used to find the free binding free energy (ΔG), utilized in our studies. In this docking tool, we exploited semi-flexible docking methods as a part of which the objective protein was kept inflexible all through the docking and the ligand was kept flexible to investigate self-assertive number of torsional degrees of freedom additionally the six spatial degrees of flexibility crossed by the translational and rotational constraints. Furthermore, AutoDock mainly utilizes Lamarckian Genetic Algorithm (LGA) to explore the best conformers. The grid separating was set to 0.375 Å and for every molecule, a most extreme of 10 docking runs were executed [14]. The energy assessment was set to 2.5×10 5 with a maximal of 27000 formations. Moreover, mutation rate and cross over were placed to 0.02 and 0.8 individually. The top-positioned compliances were examined utilizing LIGPLOT system to comprehend the pervasiveness of intermolecular connections in the complex structures. The van der Waals collaboration between the ligand and the protein, and also with the hydrophobic impact, the hydrogen bonds between the ligand and the protein, which are the main impact of deformation and these formed impacts of the translational and rotational entropy loss in the binding procedure, individually with the association of the protein and the interest of ligand. In speculation of regulating the activity of TGF- β signaling targets, we explored the interaction between Smad2, GATA2, and MAFG and TGF-β by molecular docking studies. In our study, molecular docking analysis was carried out to find out the molecular interaction of the Imiquimod, a synthetic compound in the active site of proteins such as Smad2, GATA2, and MAFG by using Grid-Based Ligand Docking with Energetic (GLIDE) from Autodock 4.2. To investigate, whether the compound that can strongly fit into the DNA binding domain, crystal structure of the targeted proteins which are downstream targets of TGF-β were used for this study. Prior to the molecular docking simulation, the binding site of proteins Smad2, GATA2, and MAFG were analysed and stated the receptor grid generation panel that generated the grid map for the receptor. A scaling factor of 1.0 was The ligands were docked using Glide XP docking protocol and it was determined all reasonable orientation for each low-energy conformer in the designated binding site. During this process, the torsional degrees of the ligand were relaxed, though the protein conformation was fixed. During the docking process, Glide that performs a complete search of conformational, orientational, and positional space of the docked ligand molecule and also shows the individual scoring function that was used to select the best conformation of the ligand molecules. Bond order of both ligand and protein were adjusted and minimized up to 0.30 Å RMSD. The OPLS-2005 force field was used for the refinement of docking including torsional and rigid-body movements of the ligand.

**Figure 1:**
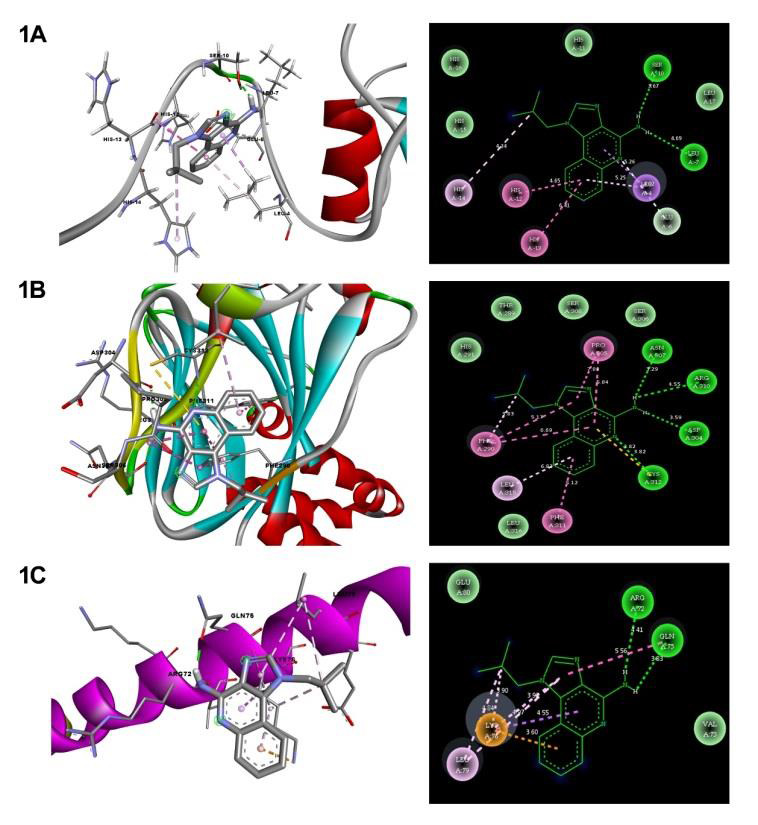
Molecular Interaction of Imiquimod with A) Smad2, B) GATA2, and C) MAFG

The molecular docking efficacy of imiquimod with TGF- β signaling targets were summarized with the binding affinities of the best pose for each interaction in Table 1. The results showed the higher binding energy of imiquimod with TGF- β signaling targets (Smad2 = -7.88 kcal mol -1, GATA2 = -5.96 kcal mol-1, and MAFG = -4.2 kcal mol -1) as mentioned in Table 1. As Figure 1A-C, shows the 3D and 2D structural representation of molecular docking analysis revealed that hydrogen bonds were formed between the imiquimod drug and the active sites of the TGF- β signaling targets residues at HIS12, GLU6, and LEU7 for SMAD2, PHE311 and LYS312 for GATA2, and LYS76, ARG72 for MAFG.

Docking analysis of these interaction results showed that the selected imiquimod drug has an efficient binding with TGF- β downstream targets target proteins Smad2, GATA2, and MAFG. From these docking results, Imiquimod found significant association of binding affinities with targets of TGF-β signaling target may results the inhibition of invasion and metastasis. So, from these results, imiquimod an oral drug might be a potential lead drug for targeting the epithelial to mesenchymal transiting pathways might lead to a better therapeutic outcome in cancer tumorigenesis.

## Conclusion:

We report the molecular docking analysis for oral cancer drug, Imiquimod with TGF-β signaling pathway targets such as Smad2, GATA2, and MAFG which are mainly plays a crucial role in regulation of Epithelial Mesenchymal Transition (EMT) in cancer cells. These docking results suggests a significant association of binding affinities between the interaction of Imiquimod and TGF-βbeta; signaling targets (Smad2, GATA2, and MAFG) which are mainly involved in the metastatic property in cancer cells. Overall, we conclude from this study was targeting the TGF-βbeta; signaling downstream with imiquimod might be a better targeting option for oral carcinoma.

## Figures and Tables

**Table 1:** Molecular docking results

**Drug**	**Proteins**	**Kcal/mol**	**Residues**
	SMAD2 (PDbID: 1KHX)	-7.88	HIS12, GLU, LEU7
			
Imiquimod (CID-57469)	GATA2 (PDB ID:509B)	-5.96	PHE311, LYS312
			
	MAFG (PDB ID:7X5E)	-4.2	LYS76, ARG72

## References

[1] Petersen PE (2005). Comm Dent Oral Epidemiol..

[2] Khandekar SP (2006). Ind J Community Med..

[3] Singh A, Ladusingh L. (2014). PLoS One..

[4] Khan Z (2017). Nicotine Tob Res..

[5] Ram H (2011). J Maxillofac Oral Surg..

[6] Navi D, Huntley A (2004). Dermatol Online J..

[7] Schön MP, Schön M. (2007). Br J Dermatol..

[8] Schön M (2003). J Natl Cancer Inst..

[9] Yingling JM (2004). Nat Rev Drug Discov..

[10] Bierie B, Moses HL. (2006). Nat Rev Cancer..

[11] Breuhahn K (2006). Oncogene..

[12] Saikia S, Bordoloi M. (2019). Curr Drug Targets..

[13] Chaitanya GV (2010). J Cell Commun Signal..

[14] Halgren HA (2004). J of med chem.

